# Recanalization of port-superior mesenteric vein thrombosis with long-term anticoagulant therapy after failed early anticoagulant therapy

**DOI:** 10.1186/s40792-024-01948-0

**Published:** 2024-06-20

**Authors:** Ichiro Okada, Masahiro Hagiwara, Hisashi Yoneyama, Saeko Kohara, Yokobori Shoji

**Affiliations:** 1 Department of Emergency and Critical Care Medicine, Nippon Medical School Hospital, 1-1-5, Sendagi, Bunkyo-Ku, Tokyo, 113-8603 Japan; 2 Department of Surgery, Sapporo Higashi Tokushukai Hospital, 3-1, North-33, East-14, Higashi-Ku, Sapporo, Hokkaido 065-0033 Japan; 3 Department of Critical Care Medicine and Trauma, National Hospital Organization Disaster Medical Center, 3256, Midoricho, Tachikawa, Tokyo 190-0014 Japan

**Keywords:** Bowel resection, Follow-up, Computed tomography, Thrombophilia, Collateral vessels

## Abstract

**Background:**

Anticoagulant therapy with heparin is the first-line treatment for acute mesenteric vein thrombosis and is effective in improving outcomes. Conversely, patients with failed early anticoagulant therapy occasionally develop bowel infarction requiring surgery. The efficacy of long-term anticoagulant therapy on recanalizing mesenteric vein thrombosis in patients with failed early anticoagulant therapy remains unclear. Herein, we report a patient who achieved recanalization of port-superior mesenteric vein thrombosis treated with anticoagulant therapy for 10 years after failed early anticoagulant therapy, followed by bowel resection.

**Case presentation:**

A 38-year-old male patient visited an outpatient clinic due to acute exacerbation of abdominal pain that had persisted for a month. He was diagnosed with port-superior mesenteric vein thrombosis on contrast-enhanced computed tomography (CT) scan and was transferred to our institution. Although he presented with abdominal pain, his respiration and circulation were stable upon hospital arrival. Anticoagulant therapy with heparin was started, and the patient was admitted to the intensive care unit. However, the patient’s abdominal pain worsened, and he began to develop signs of peritonitis. Repeat CT scan revealed bowel infarction. Thus, the patient underwent bowel resection 6 h after admission. The initial surgery was completed with open abdomen management. Bowel anastomosis was performed on the second-look surgery on the first postoperative day. Finally, the abdomen was closed on the third postoperative day after confirming the absence of bowel ischemia progression. The patient had prolonged impaired bowel function with paralytic ileus, but was discharged on the 60th postoperative day. He was then diagnosed with protein C and S deficiency based on the tests performed. Anticoagulant therapy with warfarin was initiated. He also received anticoagulant therapy in the outpatient setting. The patient’s port-superior mesenteric vein thrombosis had improved gradually with warfarin during the follow-up period. At 10 years after surgery, total occlusion of the port-superior mesenteric vein was recanalized with improvement of the portal collateral vessels. In addition, no gastric or esophageal varices were observed.

**Conclusions:**

Long-term anticoagulation therapy could affect the recanalization of extensive thrombus in multiple segments in patients with mesenteric venous thrombosis.

## Background

Mesenteric venous thrombosis (MVT) accounts for < 10% of all mesenteric infarction cases [1]. Anticoagulant therapy with heparin is the first-line therapy for acute MVT and is effective in improving outcomes [2]. Conversely, patients with failed early anticoagulant therapy occasionally develop bowel infarction requiring surgery. Recanalization of MVT is dependent on the spread of the thrombus [3]. The efficacy of long-term anticoagulant therapy in recanalizing different types of MVT in patients with failed early anticoagulant therapy remains unclear. Herein, we report a patient who underwent recanalization of port-superior MVT treated with anticoagulant therapy for 10 years after failed early anticoagulant therapy, followed by bowel resection.

## Case presentation

A 38-year-old male patient visited the emergency department of an acute-care hospital because of acute exacerbation of abdominal pain that had persisted for a month. He was previously diagnosed with Wolff–Parkinson–White syndrome and was under observation due to no history of arrhythmia. Although his general condition was stable, he presented with strong spontaneous pain and tenderness at the upper abdomen. Consequently, he was admitted to the hospital and was followed-up. He underwent abdominal contrast-enhanced computed tomography (CT) scan on the next day. Results revealed an extensive thrombus from the intrahepatic portal vein to the supra-mesenteric vein without findings of thrombus in other regions or embolization in the arterial system. The patient was diagnosed with port-superior MVT and was transferred to a tertiary hospital.

**Fig. 1:**
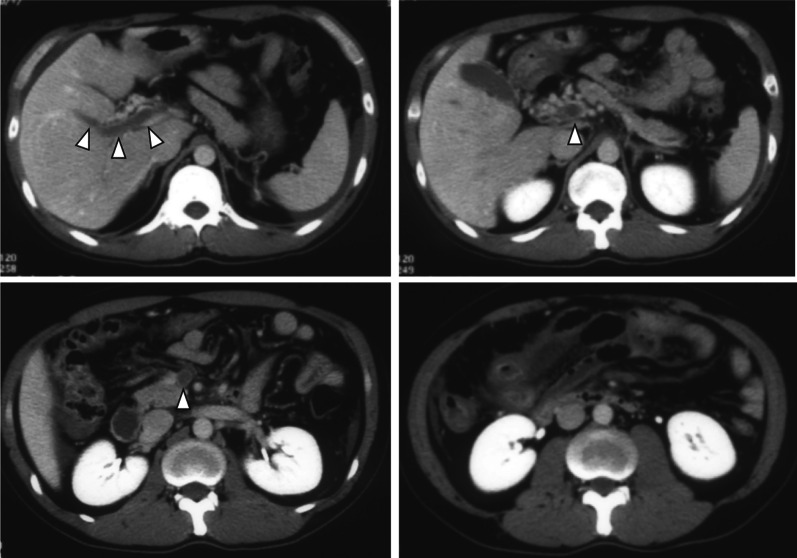
Abdominal contrast-enhanced computed tomography scan performed in the previous hospital. The thrombus extended from the intrahepatic portal vein to the superior mesenteric vein (arrows). The small intestine in the right upper quadrant was swollen but contrasted

**Fig. 2:**
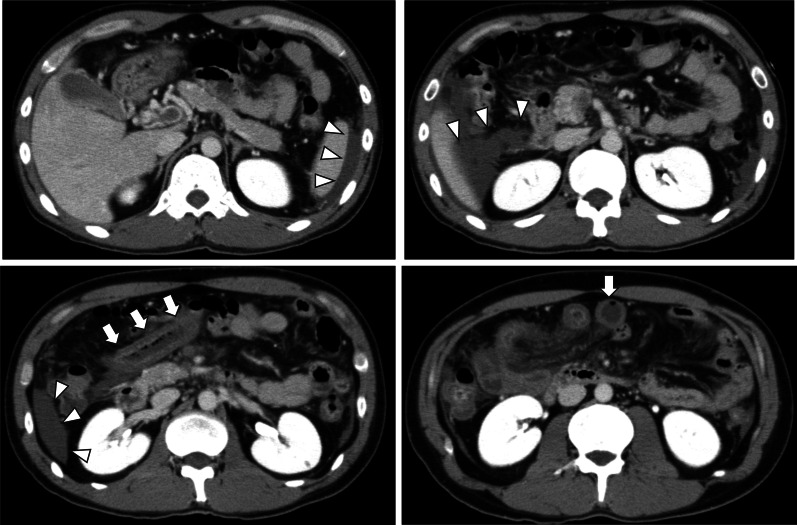
Computed tomography scan after abdominal pain exacerbation. Ascites appeared (arrows), and the contrast in the small intestine reduced (arrowheads)

Upon hospital arrival, he was alert, and his vital signs were stable (respiratory rate: 12 cycles/min, heart rate: 69 beats/min with sinus rhythm, and blood pressure: 135/74 mmHg). He complained of diffuse abdominal pain with localized mild upper abdominal tenderness. Laboratory test results were almost normal except for the following: white blood cell count, 15,700/μL; C-reactive protein level 3.2 mg/dL, prothrombin time international normalized ratio, 1.46; and D-dimmer level 21.9 μg/mL. CT scan performed in a previous hospital revealed the development of collateral blood vessels around the portal vein, and there was no reduction in the contrast effect on the bowel wall (Fig. 1). Hence, the symptoms could have been caused by chronic MVT. The patient was managed with conservative therapy particularly anticoagulation therapy using heparin in the intensive care unit. However, he complained of abdominal pain exacerbation 6 h after admission. He underwent repeat abdominal CT scan. Results revealed increased peritoneal ascites and reduced contrast effect on the small intestine wall in the right upper quadrant (Fig. 2). Small bowel infarction was strongly suspected, emergent laparotomy was performed.

During emergent laparotomy, we found approximately 500 mL of light-brown ascites in the peritoneal cavity and a 90-cm-long ileum, which was 210 cm away from the Treitz ligament to the anal side, discolored with dark brown color and sclerotic. The ileum that was evidently necrosed was partially resected. Moreover, temporary abdominal closure with the Barker method for the purpose of a second-look surgery was introduced. On postoperative day (POD) 1, a part (50-cm-long) of the small intestine was additionally resected, and anastomosis was created via hand sewing using the end-to-end Albert–Lembert suture. Abdominal compartment syndrome had been a cause of concern because the whole bowel was edematous. Open abdomen was continued for several days. Open thrombectomy was not performed due to the absence of progression in intestinal necrosis after the initial surgery. The abdomen was closed on POD 3 after bowel edema improvement.

Paralytic ileus developed after surgery and lasted long. Although anticoagulation therapy with heparin was administered during the perioperative period, oral warfarin was initiated after paralytic ileus improvement. The patient was tested for various coagulation abnormalities during hospitalization and the protein C and S activities was reduced by 20% and 46.7%, respectively. In addition, protein C antigen was reduced by 23%. However, the patient did not have a family history of thrombosis. He was evaluated by an in-house hematologist. The patient was discharged on foot on POD 60.

**Fig. 3:**
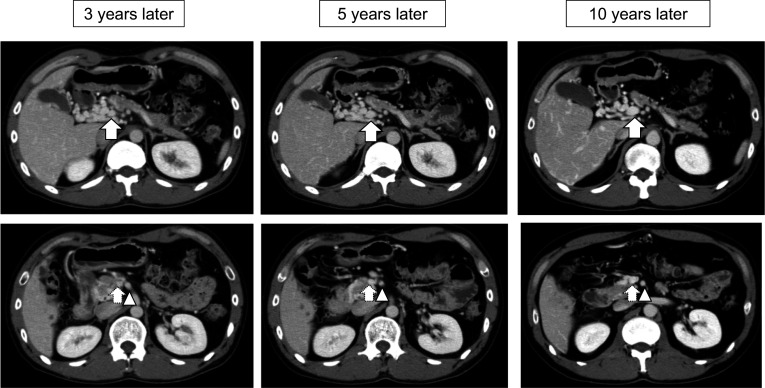
Axial view of computed tomography scans showing changes in port-superior mesenteric venous thrombus and collateral vessels over time. Thrombus in the portal vein (arrows) disappeared three years later, and the diameter of the portal vein increased over time. Collateral vessels around the portal vein improved over time. Stenosis of superior mesenteric vein (dotted arrows) gradually improved compared with the superior mesenteric artery (arrowheads)

**Fig. 4:**
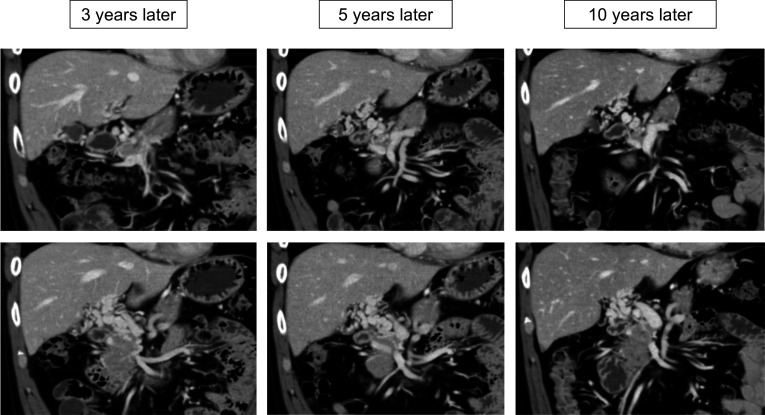
Coronal view of computed tomography scans showing changes in port-superior mesenteric venous thrombus and collateral vessels. Note the gradual improvement of collateral vessels in parallel with the gradual increase in port-superior mesenteric vein diameter

He was followed-up at the outpatient clinic every 3 months. Anticoagulation therapy with warfarin was continued. During follow-up, he was referred to the department of hematology of a university by our in-house hematologist. Protein C and S deficiency was diagnosed. Although the genetic diagnosis including family members was proposed, the patient declined the offer, and no further test was conducted. The patient was initiated on lifetime anticoagulant therapy. For follow-up evaluation of portal hypertension, esophago-gastroduodenal endoscopies and contrast-enhanced CT scans were performed on postoperative years 3, 5, and 10. Endoscopies showed neither gastrointestinal ulcer nor gastroesophageal varices. CT scans revealed gradual recanalization, and the port-superior mesenteric vein was eventually recanalized with improvement of the collateral vessels around the portal vein on postoperative year 10 (Figs. 3 and 4). During the 10-year period, the patient did not experience any event suggesting thrombosis or embolus at other sites.

## Discussion

The pathogenesis of MVT is mainly divided into three mechanisms: inflammatory process around the vein, local venous congestion due to portal hypertension, and thrombophilia [4]. Anticoagulation therapy is the mainstay therapy for MVT regardless of cause. If the therapy is introduced at an early stage, the recanalization rate is as high as > 80% [2]. However, progression to bowel ischemia requiring surgery is significantly frequent in genetic hypercoagulable disorders [5]. To prevent progression and recurrence of thrombosis, after the initial therapy, the duration of anticoagulant therapy is at least 3–6 months [2]. Lifelong anticoagulation therapy is recommended in case of thrombophilia, as in our patient, recurrence of thrombus, or thrombosis, which can lead to severe clinical complications [2]. Combined protein C and S deficiency is rare, as in our patient [6]. Case reports of adult patients with combined protein C and S deficiency who developed acute venous mesenteric ischemia are shown in Table 1 [7–10].

**Table 1:** Adult patients with combined protein C and S deficiency who developed acute venous mesenteric ischemia

Case	Age	Sex	Protein C activity (%)	Antigen (%)	Protein S activity (%)	Total antigen (%)	Free antigen (%)	Gene analysis	Thrombosis sites	Management	Recanalization	Survival	Follow-up (months)
1 [7]	57	F	36	46	N/A	30	N/A	None	Unknown	Anticoagulation therapy Bowel resection	No	Dead	None
2 [8]	52	F	20	N/A	56	N/A	N/A	None	Portal vein Superior mesenteric vein	Anticoagulation therapy Bowel resection Catheter thrombolysis	Yes	Alive	6
2 [9]	66	M	41	N/A	14	N/A	N/A	None	Portal vein	Anticoagulation therapy	Yes	Alive	6
3 [10]	63	M	54	39	59	30	N/A	None	Portal vein Superior mesenteric vein	Anticoagulation therapy	Yes	Alive	36
Our case	38	M	20	23	46.7	N/A	N/A	None	Portal vein Superior mesenteric vein	Anticoagulation therapy Bowel resection	Yes	Alive	130

Recanalization is associated with the extent of thrombus. Further, it is more frequently observed in patients with thrombosis involving the portal vein or superior mesenteric vein alone than in those with more extensive thrombosis [3]. In addition, patients with multi-segmental thrombus have a significantly lower survival [11]. A prospective study reported that the portal vein was patent in 39% of patients with anticoagulation therapy, the splenic vein in 80%, and the superior mesenteric vein in 73% after a median follow-up of 234 days [12]. Although the long-term recanalization rate remains uncertain [2, 5, 13], our case suggests that long-term anticoagulation therapy can affect the recanalization of thrombotic veins.

If there is extensive thrombus in multiple segments, thrombectomy is occasionally recommended. The role of open thrombectomy and endovascular thrombectomy is not clear [2, 4, 14, 15]. Open thrombectomy during surgery is considered an optional treatment [16]. However, intimal injury of the vessel could promote rethrombosis [2, 17].

Bleeding is the main adverse effect of anticoagulation therapy [2]. Generally, the recurrence risk without anticoagulation therapy is higher than the bleeding risk with anticoagulation therapy [11–13, 18]. The gastrointestinal tract is the main bleeding site in most cases, and the presence of gastroesophageal varices is a predictor of major hemorrhage [12, 13, 18]. Routine follow-up of gastrointestinal ulcer and gastroesophageal varices via endoscopies can be beneficial in reducing major bleeding. In our case, routine esophago-gastroduodenal endoscopy showed no findings of ulcer or varices development.

## Conclusion

Herein, we present a patient who achieved recanalization of port-superior mesenteric vein thrombosis with long-term anticoagulant therapy after failed early anticoagulant therapy. Long-term anticoagulation therapy could affect the recanalization of extensive thrombus in multiple segments in patients with MVT.

## Data Availability

Not applicable.

## Abbreviations

CT: Computed tomography
MVT: Mesenteric venous thrombosis
POD: Postoperative day
